# Parameter identifiability and model selection for partial differential equation models of cell invasion

**DOI:** 10.1098/rsif.2023.0607

**Published:** 2024-03-06

**Authors:** Yue Liu, Kevin Suh, Philip K. Maini, Daniel J. Cohen, Ruth E. Baker

**Affiliations:** ^1^ Mathematical Institute, University of Oxford, Oxford, UK; ^2^ Department of Chemical and Biological Engineering, Princeton University, Princeton, NJ, USA; ^3^ Department of Mechanical and Aerospace Engineering, Princeton University, Princeton, NJ, USA

**Keywords:** parameter identifiability, model selection, profile likelihood, cell invasion, reaction–diffusion

## Abstract

When employing mechanistic models to study biological phenomena, practical parameter identifiability is important for making accurate predictions across wide ranges of unseen scenarios, as well as for understanding the underlying mechanisms. In this work, we use a profile-likelihood approach to investigate parameter identifiability for four extensions of the Fisher–Kolmogorov–Petrovsky–Piskunov (Fisher–KPP) model, given experimental data from a cell invasion assay. We show that more complicated models tend to be less identifiable, with parameter estimates being more sensitive to subtle differences in experimental procedures, and that they require more data to be practically identifiable. As a result, we suggest that parameter identifiability should be considered alongside goodness-of-fit and model complexity as criteria for model selection.

## 1. Introduction

Partial differential equation (PDE) models have been widely employed across many areas of biology as a means to understand mechanisms driving observed behaviours, as well as for making predictions of future behaviours. The complexity of a typical biological system means that often it is not clear which mechanisms should be incorporated into a PDE model, to what detail they should be described, and what functional form they should take. As a result, there can be multiple models proposed to describe the same system. For example, a variety of growth models has been proposed and adapted to describe the growth of tumours and coral reefs, and microbial growth [1–3], and their advantages and disadvantages have been extensively studied. To be able to both make accurate predictions for unseen scenarios and elucidate underlying mechanisms, it is crucial to be able to conduct model selection.

Criteria for model selection have mostly focused on balancing model complexity with the ability of a model to accurately reproduce experimental observations. Typical examples are the Akaike information criterion (AIC) and Bayesian information criterion (BIC) [4,5]. In this work, we will show that attention should also be paid to the issue of parameter identifiability in the process of model selection, which is ignored by information criteria. Parameter identifiability refers to the extent to which the parameters of a model can be accurately estimated from available data. Non-identifiable models can give rise to misleading conclusions about the nature of the underlying mechanisms, as well as inaccurate predictions. As such, it is important to ensure that model selection considers identifiability.

In this paper, we will explore the connections between model selection and parameter identifiability through the use of *in vitro* experimental models of collective cell invasion and four related candidate PDE models to describe them. We will use profile likelihoods for identifiability analysis, and investigate the impact of model complexity, data resolution and experimental design, on parameter identifiability.

### 1.1. Parameter identifiability

The issue of parameter identifiability revolves around the question of whether it is possible to use available data to accurately estimate model parameters that describe the underlying mechanisms driving the biological system described by the model, accounting for observational errors. This inquiry revolves around two primary notions of identifiability found in the literature. The first notion is structural identifiability, which refers to the ability to uniquely determine the values of the model parameters given an infinite amount of data [6]. In the context of PDE models, this is equivalent to ensuring that distinct sets of parameter values do not yield identical solutions. While several methods exist for determining structural identifiability for ordinary differential equation (ODE) models [7,8], methods for PDE models are restricted to specific classes of models, such as age-structured PDEs [9] or systems of linear reaction–advection–diffusion equations [10].

This paper, however, focuses on the more pragmatic notion of practical identifiability. A model is considered practically identifiable if the parameters can be confidently identified using available data [6]. Practical identifiability is a stronger condition than structural identifiability, and its focus on the available data makes it more relevant for real-world applications. The main distinction between the two notions is that structural identifiability can be formulated as a property of the PDE model itself, while practical identifiability is a property of the combination of the PDE model, the error model and data [8]. In this paper, we will define a parameter to be practically identifiable if the 95% confidence region obtained using profile likelihoods for this parameter is a finite, strict subset of the parameter’s domain and the profile-likelihood function is unimodal. Moreover, for parameters that satisfy the above definition, we call a parameter more (or less) identifiable if its confidence interval is narrower (or wider) than another parameter. Note that identifiability is dependent on the parametrization of the model, and in some cases it might be possible to eliminate structural or practical non-identifiability by re-parametrizing [11,12]. In this paper, for simplicity and to ensure interpretability of the biological mechanisms, we consider the models as they are posed.

Parameter identifiability directly affects the reliability of model predictions and the ability of a mechanistic model to precisely pin down biological mechanisms, since non-identifiability may lead the modeller to place confidence in a set of parameter values that, while able to produce model solutions close to experimental data under the conditions in which the experiments were conducted, may lead to model predictions that diverge from the behaviour of the biological system under a different set of conditions.

As practical parameter identifiability is dependent on both the model and data, our investigation will focus on both of these aspects. From a data-oriented perspective, we will investigate how the quality and quantity of data impacts parameter identifiability. Specifically, we will explore the extent to which experimental design, data analysis and processing impact parameter identifiability. The results from these investigations will provide guidance for the design of experiments, as well as for data collection and processing procedures, for the purpose of improving identifiability. We will also examine the relationship between data resolution and parameter identifiability.

### 1.2. Model selection

When we build a model for a given biological system, it is often difficult to determine the appropriate level of complexity, more specifically, which mechanisms to include, and to what level of detail, and which mechanisms to simplify or neglect. When the purpose of the model is to investigate a particular mechanism, it is sensible to choose a model that revolves around that mechanism [13], which provides a lower bound on complexity. Guidelines from [1] argue that, while phenomenological models are acceptable for making predictions, a model built for the purpose of understanding the biological system should focus on mechanisms that can be concretely derived from the biology. This provides an upper bound on complexity, but in between there remains room for choice.

Increasing the level of complexity of a model by either including additional mechanisms, or using more parameters to fine-tune the description of the existing mechanisms, will potentially result in a model that is capable of fitting the given data more accurately. The downside of a more complicated model, besides over-fitting and making analysis more difficult, is that the model parameters often become less identifiable, since a change in one parameter can be more easily compensated for by changes in other parameters. This trade-off between complexity and identifiability leads us to consider the problem of model selection, where we aim to choose the most appropriate model from a collection of models of varying complexity.

Traditional tools used for addressing the dilemma between goodness-of-fit and model complexity include the AIC [14, ch. 3] and BIC [14, ch. 9]. These information criteria assign a score to models that reward goodness-of-fit, while penalizing model complexity. In this paper, we instead focus on using parameter identifiability as a tool for model selection, exploring the relationship between complexity and identifiability in further detail. We show that the model selected using information criteria, such as AIC or BIC, might contain parameters that are harder to identify than parameters in competing models that score less well, despite the widespread usage of these information criteria for model selection in mathematical biology, such as in cell invasion [15], HIV infection [16] and ecology [4]. The issue is not simply that AIC and BIC do not sufficiently penalize complexity, but rather, it is that they do not adequately quantify the relationship between complexity and identifiability. We propose that identifiability analysis should be performed prior to model selection, and that models that are identifiable given the data available should be favoured.

### 1.3. Identifiability in the context of cell invasion

Collective cell invasion plays a central role in development, wound healing and cancer, and there has been considerable interest in understanding and quantifying the mechanisms that drive, promote or hinder this phenomenon. In this work, we will use *in vitro* collective cell invasion as a prototypical biological system to explore the ways in which identifiability issues can arise, and how the profile-likelihood method can be used to analyse them. The data we will use come from a suite of barrier assay experiments, and we will use the Fisher equation and its generalizations as candidate models.

There is a long line of research on model fitting and parameter identification for cell invasion that leads up to this work. In an early work, Sherratt & Murray [17] used the Fisher model, and its generalization, the Porous Fisher model, to describe wound healing from a qualitative perspective. These models were fitted to data from cell proliferation assays by Sengers *et al.* [18]. Later, Treloar & Simpson [19] and Simpson *et al.* [20] showed that the Fisher model can be fitted to cell invasion data, using only the information on the location of the edge of the cell population. By contrast, Jin *et al.* [21] fitted the Fisher and Porous Fisher models to cell invasion data consisting of the cell density profile. The paper employed the maximum-likelihood method for parameter estimation, and goodness-of-fit as the main criterion for model selection, but it did not address parameter identifiability directly. In a later work, Vittadello *et al.* [22] used information on cell cycle dynamics, in addition to cell density data, to estimate model parameters. The same data were used by Simpson *et al.* [23] to explore parameter identifiability by quantifying the uncertainty in parameter estimates using both profile likelihoods and Bayesian inference.

The method we use for parameter inference, profile likelihoods, has been used in a variety of studies on identifiability analysis. It is built upon the maximum-likelihood estimator (MLE), and has often been compared with Markov chain Monte Carlo (MCMC)-based methods for Bayesian inference. MCMC methods are widely used for inferring parameters in mathematical biology [23–25]. These methods provide more detailed information on parameter values, but are more expensive to compute. Raue *et al.* [26] discussed the application of profile likelihoods to detect non-identifiable parameters, illustrated with an example in the context of ODE models. This work continued in Raue *et al.* [27], which suggests a method that combines MCMC methods and profile likelihoods to inform data collection and iteratively refine parameter estimates. A comparison between the methods of MCMC and profile likelihoods was carried out in [28] for ODE models of varying complexity. Other methods for inference include gradient matching [29,30] and approximate Bayesian computation [31]. The application of profile likelihoods in model selection was discussed by Simpson *et al.* [3], who used three different ODE models to describe coral growth, and discussed the importance of parameter identifiability in model selection. Model selection for cell invasion models has been discussed by Warne *et al.* [15], who used Bayesian methods for model fitting, and information criteria for model selection. The authors emphasized the importance of model complexity as a factor in model selection and identified BIC as the best overall criterion. However, the authors realized that information criteria cannot account for all aspects of model comparison.

### 1.4. Outline

The contribution of this paper is the investigation of parameter identifiability for multiple PDE models using profile likelihoods, and a discussion on the implications of practical identifiability in model selection. We also investigate the effects of data resolution and experimental design on parameter identifiability. Lastly, we demonstrate a link between parameter identifiability and model robustness in terms of consistency of parameter estimates, which motivates a mixed-effects view of the system. Under this view, each experimental replicate can be seen as taking a sample of the parameter values from a certain distribution, which tends to be more dispersed for less identifiable models. The rest of the paper proceeds as follows. We describe the experimental procedures for the barrier assay, as well as data processing procedures, in §2. We introduce the suite of mathematical models, as well as the profile likelihood method for parameter inference and identifiability analysis, in §3. The results are presented in §4, and we discuss their significance with respect to model selection and experimental design in §5.

## 2. Experimental methods and data

Barrier assays, also known as tissue expansion assays, are a way to observe cell invasion *in vitro*. Briefly speaking, a barrier assay involves preparing a plate with a barrier that encloses a central region which cells cannot move in or out of. Cells are planted within the central region, and then given sufficient time to proliferate to form a monolayer within the central region. The barrier is then removed, allowing the cells to propagate outward and invade the rest of the plate, which is initially absent of cells. For this work, eight experiments were carried out in total, with the first four (Experiments 1, 2, 3 and 4) using a circular barrier, and the later four (Experiment 5, 6, 7, 8) using a triangular barrier. The experiments were otherwise identical. Selected images from the experiments are shown in figure 1, along with the corresponding schematic of the experiment, and snapshots of model solutions at the corresponding time point. The detailed experimental protocols are provided in §§2.1 and 2.2.

**Figure 1.**
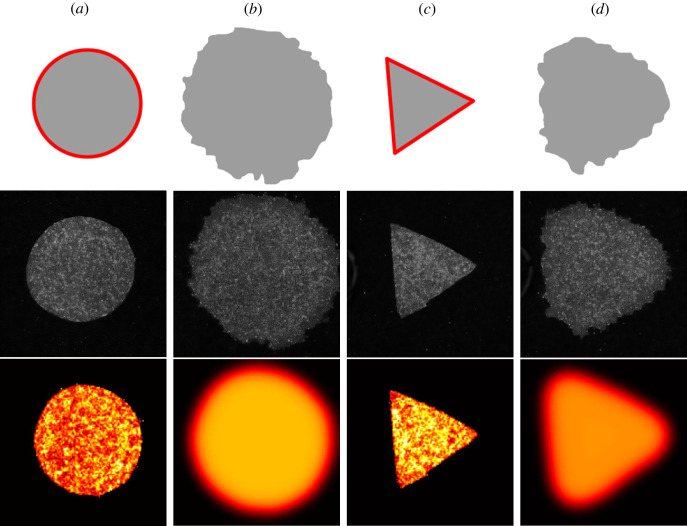
Illustration of various stages of the barrier assay. In the first row are simplified representations of the cell culture, where the grey areas represent the regions covered by cells, and the red lines represent the barriers. The second row contains images taken from the experiments. The field of view of the camera is limited to a square region of size 4380 by 4380 μm in the centre of the plate, while the plate itself extends further beyond. Since the cells tend to stay well within the field of view, except toward the very end of the experiment, edge effects are not important. The plate is sufficiently large such that the cells never reach the edge in any experiments. The third row represents snapshots from simulations of the Standard Fisher model (table 1) with the free parameters set to the MLE computed from the corresponding experiments. The colours represent cell density, with black indicating a complete absence of cells, while the cell density increases as the colour progresses from red to light yellow. (*a*) Initial condition of Experiment 1, where the initial confinement region is circular. The barrier is removed at *t* = 0 h. (*b*) The state of the experiment at *t* = 20 h. The cells have spread out to cover most of the visible domain. (*c*,*d*) Shows the initial conditions at *t* = 0 h and a later stage *t* = 20 h of Experiment 5. The experiment is similar to Experiment 1, except with a triangular barrier.

### 2.1. Cell culture

Madin–Darby Canine Kidney (MDCK-II) cells are cultured in low-glucose Dulbecco’s modified Eagle medium (DMEM) with 10% fetal bovine serum (FBS) and 1% penicillin–streptomycin. Cells were split every 1–2 days, depending on the conditions of the cells. MDCK cells were treated with TrypLE for 8 min to be detached from the tissue-culture treated (TC) plastic dish. Detached cells were 1 : 2 diluted with the culture media and centrifuged at 300*g* at room temperature for 3 min. Cell pellets were then re-suspended with culture media and seeded in the new dish.

### 2.2. Barrier assay

The TC plastic dish is coated with 50 μg ml^−1^ Collagen 4 in PBS at 37°C for 30 min. A 250 μm thick polydimethylsiloxane (PDMS) membrane cut with a Silhouette Cameo vinyl cutter was deposited on the TC plastic dish, which confines the cells to the initial region. A $2.25\times 10^{6}\;\mathrm{cells}\;ml^{-1}$ MDCK cell suspension was seeded in each stencil. The seeding volume was 0.44 μl per mm^2^ of tissue area. The TC plastic dish was incubated at 37°C for 30 min to induce cell attachment to the collagen surface. Subsequently, the dish was flooded with the culture medium, and cells were incubated for 19 h to form a confluent monolayer. Stencils were removed from the dish with tweezers at the start of the barrier assay. The expansion of tissue monolayer was imaged from this point onward every 20 min for 24 h with 4× phase objective.

### 2.3. Image analysis

From the 4× phase images, nuclei locations were reconstructed using a tool based on convolutional neural networks [32]. The local cell density was calculated by counting the number of nuclei centroids in each density measuring box. The box size is 58.4 × 58.4 μm, with 50% overlap between neighbouring boxes.

## 3. Mathematical methods

### 3.1. PDE models of cell invasion

To describe the cell invasion process observed in the barrier assay experiments, we will use the Fisher–Kolmogorov–Petrovsky–Piskunov (Fisher–KPP) equation, and its extensions, to model the evolution of cell density over time. The original Fisher model was proposed in [33], and studied in a more general context in [34]. More recently in cell biology, this model and its generalizations have been used to model a diverse range of phenomena including wound healing [35,36] and tumour growth [37–39].

We summarize the Fisher model and its generalizations as

3.1a $$\frac{\partial C}{\partial t}=\nabla \cdot (D(C)\nabla C)+f\;(C),$$

3.1b $$D(C)=D_{0}{\left(\frac{C}{K}\right)}^{\eta },$$

3.1c $$\begin{array}{r} \mathrm{and}\;\;f\;(C)=rC^{\alpha }{\left[1-{\left(\frac{C}{K}\right)}^{\gamma }\right]}^{\beta },\;0<x,y<L,\;t>0. \end{array}$$

In the context of our application, *C*(*x*, *y*, *t*) ≥ 0 represents cell density, *D*(*C*) ≥ 0 is the diffusion coefficient, and the function *f*(*C*) represents net cell proliferation, with *K* > 0 representing the carrying capacity. The parameters *D*_0_ > 0 and *r* > 0 scale the rates of diffusion and proliferation, respectively, and *α*, *β*, *γ*, *η* are non-negative ‘shape parameters’ that allow fine-tuning of *D*(*C*) and *f*(*C*). We impose zero-flux boundary conditions at *x* = 0, *L* and *y* = 0, *L*.

We consider four different models. The original Fisher model takes *D*(*C*) = *D*_0_ and *f*(*C*) = *rC*(1 − *C*/*K*), which corresponds to fixing *α* = *β* = *γ* = 1 and *η* = 0. The Porous Fisher model generalizes the diffusion term by allowing *η* ≥ 0 to be free. This model has been used to describe cell invasion and wound healing [17,18,21,24,40]. The intuitive motivation is that as cell density increases, a crowding effect will push the cells to move into less dense areas more quickly, leading to density-dependent diffusion. The theoretical properties of the model have been studied in many works, including [41,42]. The Richards model, proposed in [43], and the Generalized Fisher models both generalize the proliferation term by allowing *γ* ≥ 0 and *α*, *β* ≥ 0 to assume values other than unity, respectively. The Richards and Generalized Fisher models, among other growth laws, were analysed in the context of ODE models in [44]. We summarize the four models in table 1, and the numerical methods used for simulating these models are given in electronic supplementary material, S1.1.

**Table 1.** Summary of models considered, as special cases of equation (3.1).

model name	free parameters	fixed parameters
Standard Fisher	*D*_0_, *r*, *K*	*α* = *β* = *γ* = 1, *η* = 0
Porous Fisher	*D*_0_, *r*, *η*, *K*	*α* = *β* = *γ* = 1
Richards	*D*_0_, *r*, *γ*, *K*	*α* = *β* = 1, *η* = 0
Generalized Fisher	*D*_0_, *r*, *α*, *β*, *K*	*γ* = 1, *η* = 0

### 3.2. Parameter identifiability with profile likelihoods

For a given model, let *P* be the number of free parameters, ***θ*** denote the vector of free parameters in the model, and ***θ***_−*i*_ denote the parameter vector with *θ*_*i*_, the *i*th parameter, removed.^1^ For the Standard Fisher model, for example, *P* = 3 and ***θ*** = (*D*_0_, *r*, *K*), and ***θ***_−2_ = ***θ***_−*r*_ = (*D*_0_, *K*). Let *C*_data_(*x*, *y*, *t*) be the cell density measurements, and let *C*_model_(*x*, *y*, *t*; ***θ***) be the solution to equation (3.1) with parameter set ***θ***. We assume that the observed cell density is generated by the model with given model parameter set $\hat{\theta }$, and perturbed by independent and identically distributed (i.i.d.) observation noise at each data point that follows a Gaussian distribution with zero mean and unknown variance *σ*^2^. That is,

3.2 $$\begin{array}{rl} & C_{\mathrm{data}}(x_{i},y_{j},t_{k})=C_{\mathrm{model}}(x_{i},y_{j},t_{k};\hat{\theta })+\epsilon_{ijk}\;\mathrm{and}\\ & \;\epsilon_{ijk}∼N(0,\sigma^{2}). \end{array}$$

We acknowledge that this error model is not perfect, but nonetheless we use it for its simplicity and wide adoption in the literature. We consider ways in which the error model could be improved in the Discussion. Furthermore, let *L*(*C*_data_|***θ***, *σ*) be the likelihood function. Let θ*, σ* be the MLE. We define the normalized profile log-likelihood function *l*_*i*_(*θ*_*i*_′) for parameter *θ*_*i*_ to be

3.3 $$l_{i}(\theta_{i}^{\prime })=\max_{\theta_{-i},\sigma }[\log L(C_{\mathrm{data}}|\theta ,\sigma ){|}_{\theta_{i}=\theta_{i}^{\prime }}]-\log L(C_{\mathrm{data}}|\theta^{*},\sigma ),$$

which will be referred to simply as the profile-likelihood function for brevity. We can similarly define a bivariate profile likelihood, *l*_*i*,*j*_, as

$$l_{i,j}(\theta_{i}^{\prime },\theta_{j}^{\prime })=\max_{\theta_{-i,-j},\sigma }[\log L(C_{\mathrm{data}}|\theta ,\sigma ){|}_{\theta_{i}=\theta_{i}^{\prime },\theta_{j}=\theta_{j}^{\prime }}]-\log L(C_{\mathrm{data}}|\theta^{*},\sigma^{*}).$$

Following [3,45], we use the profile-likelihood function to define an approximate 95% confidence interval for a single variable as

$$\left\{\theta_{i}|l_{i}(\theta_{i})>-\frac{\chi^{2}(0.95;1)}{2}\approx -1.92\right\},$$

and a joint confidence region for two variables as

$$\left\{\theta_{i},\theta_{\;j}|l_{i,j}(\theta_{i},\theta_{j})>-\frac{\chi^{2}(0.95;2)}{2}\approx -3.00\right\},$$

where *χ*^2^( · ; *m*) denotes the inverse of the cumulative distribution function (CDF) of a *χ*^2^ distribution with *m* degrees of freedom. The shape of the univariate profile-likelihood curve indicates whether or not the parameter is practically identifiable. A profile-likelihood curve that is unimodal, smooth, and decays quickly away from its peak (which occurs at the MLE) suggests that the parameter is identifiable. Non-identifiability can be reflected in a profile likelihood in many different ways, such as multi-modality, slow decay away from the MLE, or a flat top.

## 4. Results

We now present the results from the identifiability analysis of the four models using profile likelihoods. A preliminary exploration with synthetic datasets, presented in electronic supplementary material, section S2, shows that profile likelihoods can recover the ground truth parameter values in the absence of model mis-specification. In §4.1, we show that all models considered are practically identifiable. Then, in §4.2, we show that slight changes to data processing procedures have a major impact on the parameter estimates for more complicated models, but not for simple models. In §4.3, we compare the profile likelihoods across multiple experimental datasets to observe a relation between model complexity and consistency of parameter estimates. Finally, in §4.4, we investigate the relationship between data resolution and practical identifiability.

### 4.1. All four models are practically identifiable

First, we show that all four models are practically identifiable. We present the profile likelihoods for Experiment 1 in figure 2, which is arbitrarily chosen as an illustrative representative for the purpose of this section. The results for the other experiments are qualitatively similar. We present the profile likelihoods for the other experiments, and the MLEs, the 95% confidence intervals, and AIC and BIC for each model for each experiment, in electronic supplementary material, section S3. Visual inspection indicates that the MLEs for all four models give rise to model predictions that match the data very well, and the residuals are small compared with the magnitude of the solution. As such, it is difficult to select between models by such visual inspection alone. A likelihood-ratio test for nested models ([46], details in electronic supplementary material, section S3) shows that the predictions of all three more complicated models are significantly better than the Standard Fisher model. We note that, in cases where the fit of all candidate models is poor, comparison with a non-mechanistic model, as carried out in [47], could point to directions where the mechanistic model could be improved.

**Figure 2.**
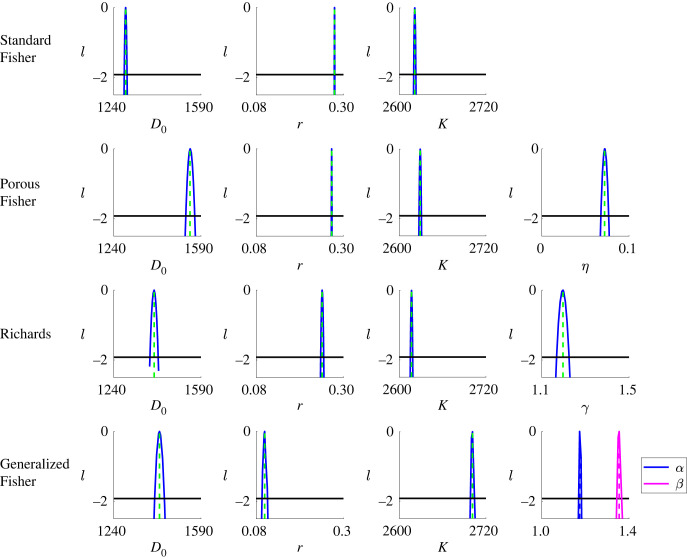
Profile likelihoods using data from Experiment 1. The vertical dashed lines mark the MLE for each parameter, and the black horizontal line at −1.92 marks the threshold for the 95% confidence interval.

Observe that in figure 2, all profile-likelihood curves are unimodal, and roughly parabolic in shape, and the confidence intervals are narrow. These observations, and similar results from the other experiments (shown in electronic supplementary material, section S3), suggest that all models are practically identifiable given available data from any one of the eight experiments. Comparing the Standard Fisher model with the more complicated models we notice that, as model complexity increases, the profile-likelihood curves broaden, and therefore the confidence intervals widen, making the parameters less identifiable. This suggests that increases in model complexity tend to lead to decreases in parameter identifiability. Intuitively, in the absence of model mis-specification, for nested models, the more complex model with (more free parameters) will be less identifiable since there is greater possibility that a change in the value of one parameter can be compensated for by changes in other parameters. However, in the presence of model mis-specification, identifiability may improve as a result of introducing additional parameters, for example, if the additional parameter rectifies a flaw in a mis-specified model. Such cases should happen rarely in practice, since ideally such a flaw should be identified prior to the model selection stage of a study. Both residual analysis and posterior predictive checks provide effective tools for detecting model mis-specification. Residual analysis is computationally cheaper, does not rely on assumptions on a prior distribution for parameter values, and complements likelihood-based methods well, whereas a posterior predictive check is natural to implement after performing Bayesian inference.

### 4.2. Complicated models are more sensitive to data processing

We now turn to the question of whether the way we process the data impacts parameter identifiability. In the case where the barrier is circular, the cell population remains approximately radially symmetric throughout the experiment, as expected. In this case, we can average the data radially to obtain a reduced dataset ${\tilde{C}}_{\mathrm{data}}(\rho ,t)$. It is tempting to work with this reduced dataset instead of the full dataset, since it makes model simulation, and therefore parameter inference and identifiability analysis, computationally much cheaper.

In figure 3, we show the profile likelihoods computed using the radially averaged dataset from Experiment 1. Comparing figures 2 and 3, we notice that, in general, the profile-likelihood curves are broader for the radially averaged dataset compared with the full dataset. This is expected, since the full dataset has many more data points. A more surprising observation is that, while the MLEs for the parameters in the Standard Fisher model remain consistent between the two datasets, the MLEs for the other three models are much less consistent. These observations show that the more complicated models are more sensitive to the way the data are processed or represented.

**Figure 3.**
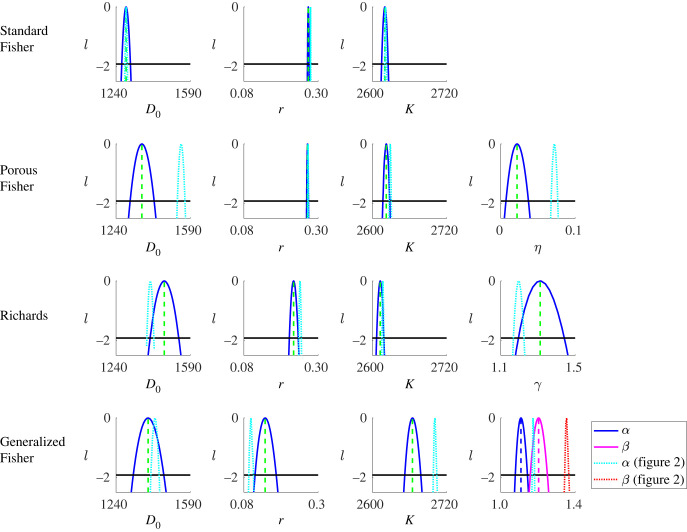
As per figure 2, except using the radially averaged dataset ${\tilde{C}}_{\mathrm{data}}(\rho ,t)$. The axes are the same as in figure 2. The profile-likelihood curves from figure 2 are included here as dotted lines for ease of comparison. Notice that the profile-likelihood curves are broader compared with figure 2, and the location of the MLE for the Porous Fisher, Richards and Generalized Fisher models are shifted, but remain virtually unchanged for the Standard Fisher model.

In the absence of noise or model mis-specification, and an abundance of data, consistent MLEs will be obtained regardless of data representation, since applying the same transformation (in this case, radial averaging) to the data and the model will not change the fit. However, when the data are noisy and/or the model is mis-specified, this will generally not be the case, as we see here. There are several reasons for this. First, we have implicitly assumed within our model that the transform is applied to the (noiseless) data, then additive noise is applied to the transformed data. However, in reality, the transform is applied to the noisy data, and so the radially averaged dataset is not equivalent to the original full dataset. Second, radial averaging smooths the cell density. In particular, near the edge of the tissue where there are deviations from radial symmetry clearly observable in the data (figure 4) we see a smoothing of the leading edge density profile. The more complicated models have increased flexibility to fit the leading edge data, which drives changes in the MLEs. As a result, we suggest that in cases where process noise is significant we should favour a simpler model, which is more robust to overfitting.

**Figure 4.**
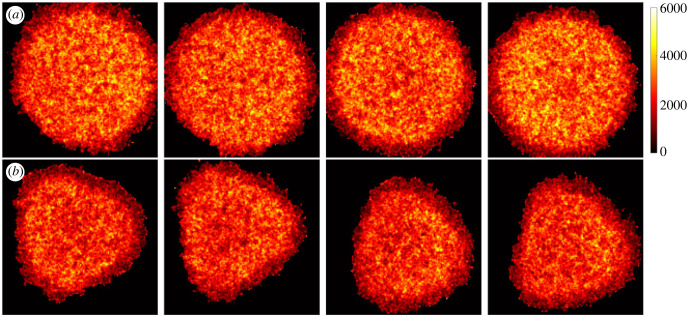
The cell density in the eight experiments at the final time point. (*a*) Experiments 1–4, (*b*) Experiments 5–8. Notice that although the final states are qualitatively similar across experiments with the same initial conditions, there are quantitative differences. For example, the mean cell density for Experiments 1–4 lies between $2413\;\mathrm{and}\;2552\;{\mathrm{cells}\;\mathrm{mm}}^{-2}$, which is higher than the $2064-2138\;{\mathrm{cells}\;\mathrm{mm}}^{-2}$ observed in Experiments 5–8.

### 4.3. Consistency of parameter estimates across experimental replicates indicates practical identifiability

By examining and comparing the profile-likelihood results across all experimental replicates, we found that the parameter estimates are less consistent for the more complicated models compared with the simpler Standard Fisher model, in the sense that the variances of the eight MLEs of analogous parameters are higher for the more complicated models. In figure 5, we present the confidence intervals for all parameters in the four models found across the eight experimental replicates, except for the Generalized Fisher model, where we only present the results from the first four experiments.^2^

**Figure 5.**
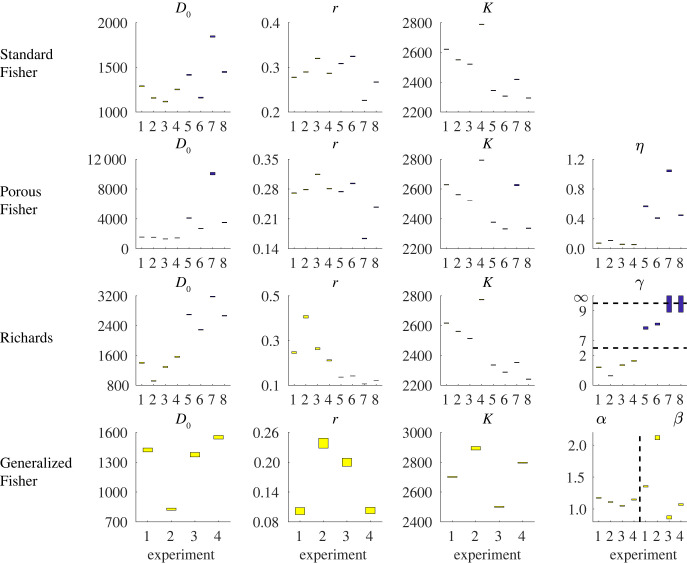
Comparison of the 95% confidence intervals for the parameters in the four models obtained using profile likelihoods on the eight experimental datasets. The confidence intervals are represented as ranges by vertical bars. The experiments with circular initial conditions (Experiments 1–4) are labelled in yellow, while the ones with triangular initial conditions (Experiments 5–8) are labelled in blue. Note that some vertical axes do not start from zero. Note that the horizontal dashed lines in the figure for *γ* in the Richards model indicate breaks in *y*-axis.

For all parameters, the widths of the confidence intervals for individual experiments are much narrower compared with the range of estimates across all experiments. For example, the widths of the confidence intervals for *D*_0_ in the Standard Fisher model are of the order of 10 μm^2^ h^−1^, whereas the MLEs for *D*_0_ for Experiments 3 and 7 differ by around 700 μm^2^ h^−1^. The confidence intervals for the same parameter estimated from different experiments rarely overlap; these variations in parameter estimates reflect the high variability commonly observed in biological processes (see figure 4). These results suggest that it may instead be appropriate to model the system using a fixed-effects or a mixed-effects framework so that parameters can vary across biological replicates. In addition, we note that the discrepancies could, in part, arise from spatial and temporal auto-correlations in the noise (figure 4 and [48])—see the Discussion for more details on this point.

We also find that the parameter estimates for the more complicated models seem to depend on the initial conditions, with the experiments with circular initial conditions yielding significantly different parameter estimates compared with the ones with triangular initial conditions. For the rest of the section, we will review these differences in detail, and discuss possible biological explanations.

For the Standard Fisher model, the MLEs for the parameters show only a mild degree of variability between the experiments. The difference in initial conditions does not lead to significant differences in the estimates of *D*_0_ and *r*,^3^ while the estimate for *K* tends to be slightly lower for the experiment with triangular initial conditions as opposed to circular initial conditions.^4^

While the variability of parameter estimates in the Standard Fisher model across experiments is modest, it is much larger in the other models: for example, the standard deviations for the MLEs for *D*_0_ across the experiments are 2937.8 μm^2^ h^−1^ for Porous Fisher, 812.9 μm^2^ h^−1^ for Richards and 320.4 μm^2^ h^−1^ for the Generalized Fisher models, compared with 238.3 μm^2^ h^−1^ for Standard Fisher, although this comparison might not be entirely fair for the Porous Fisher model, since the diffusion coefficient is density-dependent, hence the interpretation of *D*_0_ is different. Similar results hold for *r*, while the variability for *K* is approximately the same across the four models.

For the Porous Fisher model, the experiments with circular initial conditions (Experiments 1–4) estimate a very small *η*, well below 0.2, suggesting that there is little evidence for nonlinear diffusion in the dynamics, and the estimates for the other parameters (*D*_0_, *r*, *K*) are relatively similar to their estimates in the Standard Fisher model. However, the experiments with triangular initial conditions (Experiments 5–8) estimate a much larger *η*, along with a higher *D*_0_,^5^ suggesting that nonlinear diffusion effects are important. Since the speed of the propagation of the cell colony (which can be identified as the speed of the travelling wave in the model solutions) does not vary much between the experiments, and an increase in *η* corresponds to a decrease in wave speed, the estimated values for *D*_0_ and *r* are much higher for these experiments to compensate for a higher *η*. This compensation is reflected by the slanted ridge seen in the bivariate likelihood function for *D*_0_ and *η*, shown in figure 6. Although both parameters are identifiable in this case, such correlations in parameter values are a typical source of non-identifiability.

**Figure 6.**
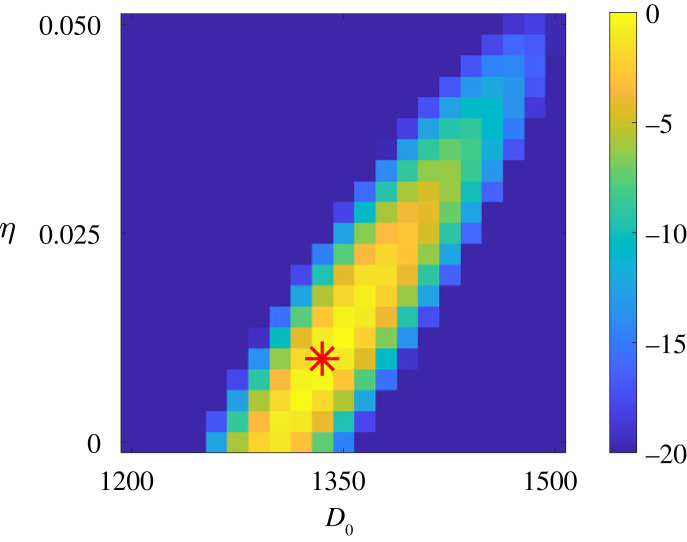
Bivariate profile-likelihood function for *D*_0_ and *η* in the Porous Fisher model represented as a heat map, computed using the dataset from Experiment 1. The location of the MLE is indicated by the red star. The slant of the bivariate likelihood contours indicates that the two parameters can compensate for each other.

We see a similar story for the Richards model. Observe that in the third row of figure 5, there seems to be a clear dependence of the estimated parameter values on the experimental initial conditions. Experiments 1–4 (circular initial conditions) in general give lower values for *D*_0_, *r* and *γ*, and higher values for *K*, compared with Experiments 5–8 (triangular initial conditions). Especially striking is the case of *γ*, where Experiments 1–4 give estimates in the range of 0.6–1.7, whereas Experiments 5–8 give estimates either around 8–9, or above 9 (we use *γ* = 9 as an upper bound for calculations when searching for the MLE to ensure numerical stability). The Richards model is also the only model that has profile likelihoods which do not have a roughly parabolic shape. For Experiments 5 and 6, the profile likelihoods for *γ* are bimodal (but the peaks are close together, and the valley between them lies above the −1.92 threshold, so the confidence region remains a connected interval). This bimodality can also be seen in the profile likelihoods for the other parameters, but is much less prominent. For Experiments 7 and 8, the profile-likelihood functions for *γ* appear to be monotonically increasing for large *γ* until the imposed upper bound at *γ* = 9, suggesting that the MLE is either very large or tends to infinity.

A high *γ* (say, above 7) means that the proliferation function *f*(*C*) in equation (3.1) is nearly linear for 0 ≤ *C* < *K* except for *C* near *K*, and it abruptly drops to zero at *C* = *K*. An even higher *γ* makes little difference to the shape of *f*(*C*), and therefore has little effect on the model solution. For this reason the Richards model is known to be locally structurally non-identifiable in the high *γ* regime [23]. This explains the divergence to infinity of the confidence intervals for *γ* for Experiments 7 and 8.

It is more difficult to explain why the experiments with triangular initial conditions suggest a very large *γ*, hence nearly linear proliferation, whereas the experiments with circular initial conditions suggest *γ* is relatively close to one, reflecting dynamics closer to logistic growth. However, there are previous studies making similar observations. In [21], the authors showed that parameter estimates for similar experiments and models depend strongly on the initial cell density, when the shape of the initial cell population is kept constant. By contrast, however, Jin *et al.* [49] found that the shape of the initial cell density profile does not seem to have a major effect on the estimates for the parameters in the Standard Fisher model, which agrees with our observations.

The variability of the parameter estimates in the Porous Fisher and Richards models, and their dependence on initial conditions, is a concern, since we expect the experimental replicates to represent the same biological processes. The observed variability could be due to the fact that the more complicated models have greater propensity to overfit to noise, as we highlighted earlier. Furthermore, note that the high inherent variability in biological processes means that the dynamics driving cell behaviour in each replicate are slightly different. Tissue mechanics offers another biological explanation for the dependence of dynamics on initial conditions. In an empirical study, Ravasio *et al.* [50] demonstrated that edge geometry has a significant influence on the rate of wound closure. This effect arises from the exertion of force on the cells by supra-cellular actomyosin cables around the wound edge which, in turn, is regulated by the underlying geometry. The Standard Fisher model might be too simplistic to capture the effects on cell behaviour due to geometry, and therefore could be mis-specified. The more complicated models are able to reflect this effect to some extent and therefore partially mitigate against model mis-specification, although without explicitly incorporating tissue mechanics and geometry into the model. More specifically, the curvature of the tissue edge changes more dramatically in the experiments with triangular initial conditions (the straight edges evolve to rounded, convex edges, while the sharp corners are rounded out) compared with the experiments with circular initial conditions (where the curvature only slightly decreases as the circle enlarges). Since cell density is also increasing throughout the experiment, the curvature effect might be reflected in the Porous Fisher model as an apparent stronger dependence of the diffusion coefficient on cell density for the experiments with triangular initial conditions, and therefore a higher estimated *η*.

For the Generalized Fisher model, the computations for the profile likelihoods did not finish within a reasonable time limit for Experiments 5–8, so we only present the parameter estimates for Experiments 1–4 in the last row in figure 5. For Experiments 5–8, the optimization procedures struggled much more to find the correct global maximum compared with Experiments 1–4. The likelihood landscape in this case is much more complex, and all the optimization algorithms we tried repeatedly converged to ‘wrong’ local minima, despite many iterations of restarts with improved initializations and the implementation of alternative optimization algorithms. This is because, for reasons that we do not yet fully understand, there are many more (deeper) local minima in the likelihood landscape when the initial condition is a triangle, perhaps due to a lack of symmetry in the data. This is a difficult computational problem for MCMC methods as well, such as Metropolis–Hastings, where we found that Markov Chains failed to converge after a very large number of iterations, compared with the number of iterations required for the simpler models.

### 4.4. Model complexity is limited by data resolution

In order to investigate the relationship between parameter identifiability and data resolution, we repeat the profile likelihood calculation on the reduced dataset from Experiment 1, but progressively down-sample the data to reduce temporal resolution by using only an equally spaced subset of the 77 time slices in our data. We observe three different ways in which the profile-likelihood curves change qualitatively as data resolution decreases. We present the profile likelihood for one parameter to illustrate each case, and leave the rest of the parameters to electronic supplementary material, section S4. In the first case, the peak of the profile likelihood curve (i.e. the MLE) remains mostly in the same location, while the curve itself broadens, but nonetheless remains unimodal. This is illustrated by *D*_0_ in the Standard Fisher model, shown in figure 7*a*. In this case, despite increases in the uncertainty in parameter estimates, the parameter remains practically identifiable even at the lowest data resolution we considered. This is the case for all parameters in the Standard Fisher model, as well as *K* in all models, and *D*_0_ and *r*, except in the Generalized Fisher model.

**Figure 7.**
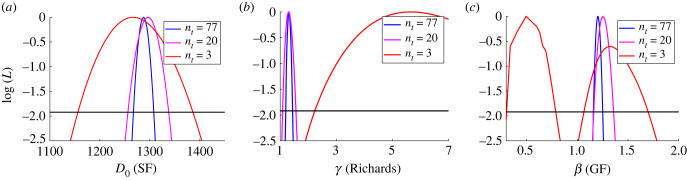
Comparison of profile-likelihood curves calculated using progressively down-sampled data from Experiment 1, for (*a*) *D*_0_ in the Standard Fisher model, (*b*) *γ* in the Richards model and (*c*) *β* in the Generalized Fisher model. *n*_*t*_ denotes the number of time points used. The blue curve corresponds to *n*_*t*_ = 77, i.e. the profile likelihood calculated with all available data, with a temporal resolution of Δ*t* = 20 min. The pink curve corresponds to *n*_*t*_ = 20 and Δ*t* = 80 min, and the red curve corresponds to *n*_*t*_ = 3 and Δ*t* = 12 h. The black horizontal line at −1.92 is the threshold for the confidence interval. Note that in (*b*), the red curve remains above the threshold as *γ* → ∞, and we chose to truncate the plot at *γ* = 7 for ease of visualization.

In the second case, as illustrated by *γ*, a shape parameter in the Richards model in figure 7*b*, the location of the MLE dramatically changes, and the shape of the profile-likelihood curve changes in such a way that the confidence interval becomes infinite, making *γ* non-identifiable in the case where data resolution is low. Interestingly, *γ* is the only parameter that exhibits this behaviour. The final case is shown in figure 7*c*, where the profile likelihood curve for *β* in the Generalized Fisher model becomes bimodal when the data resolution is sufficiently low. *D*_0_, *r* and *α* in the Generalized Fisher model also exhibit this behaviour.

Theoretically, we expect the width of the confidence interval for a parameter *θ*, denoted Δ*θ*, to be proportional to $1/\sqrt{N}$, where *N* is the number of data points. We compare the theoretical and calculated values of Δ*θ* in figure 8. Observe that for *D*_0_ in the Standard Fisher model, the calculated Δ*D*_0_ remains close to the theoretical prediction as *N* → 0, while Δ*γ* and Δ*β* deviate significantly from the theoretical prediction as *N* → 0.

**Figure 8.**
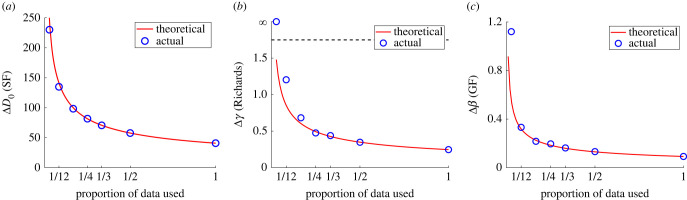
The widths of the confidence intervals of three selected parameters plotted against the proportion of data used, for (*a*) *D*_0_ in the Standard Fisher model, (*b*) *γ* in the Richards model and (*c*) *β* in the Generalized Fisher model. Here, using a proportion of 1/*m* of data means that only one in every *m* images was used, so $n_{t}=\lceil 77/m\rceil$. The blue circles represent the widths of the confidence intervals calculated by finding the intersection between the profile likelihood curves and the threshold −1.92, while the red line is proportional to $1/\sqrt{n_{t}}$ and normalized so that it goes through the point representing the case where all data were used.

These observations show that parameter identifiability is limited by data resolution. There is a model-dependent minimum amount of data required for the model to be practically identifiable, and this minimum increases as model complexity increases. Therefore, model selection should be tied to the amount of data available, and as a criterion for choosing a model, we should only use models for which we have sufficient data to make them identifiable.

## 5. Discussion and future work

In this paper, we have carried out an in-depth study of practical parameter identifiability for four related PDE models of cell invasion using the profile likelihood method. We have shown that, given sufficient data, the univariate profile likelihoods of all model parameters are unimodal, with the corresponding confidence intervals relatively narrow (with the exception of the Richards model for datasets with triangular initial conditions), suggesting that the parameters are practically identifiable. Moreover, results obtained using synthetic data (electronic supplementary material, section S2) demonstrate that the profile-likelihood method is capable of recovering the true parameter values in the absence of model mis-specification.

We explored the effects of several aspects of data quantity and quality on parameter identifiability. First, by comparing the profile likelihoods obtained using the full density profile with the ones obtained using the radially averaged density profile, we found that the parameter estimates obtained for the Standard Fisher model are approximately the same for the two cases, but differ considerably for the more complicated models. This result suggests that simpler models are more robust against subtle changes in data representation. For such models, it is therefore safe to perform inference using the radially averaged dataset to reduce computational costs, whereas the full dataset should be used for parameter inference for the more complicated models, which are more sensitive to changes introduced by data processing. If process noise is believed to be significant, then simpler models should be preferred, since they tend to be more robust against overfitting to the process noise.

We have shown that, for any parameter, the confidence intervals obtained for a single experiment are narrow relative to the spread of the estimated values for the same parameter across experiments. There are two plausible explanations for this. First, due to the high inherent variability in biological systems, the true dynamics behind each experiment replicate may be slightly different. This result suggests that a nonlinear mixed-effects framework may be appropriate. In this view, each experimental replicate corresponds to a parameter set sampled from a specified distribution. However, significantly more experiment replicates will be required to determine a suitable form for these random effects. Furthermore, since the shape of the barrier has a consistent effect on some parameters, it is reasonable to include the shape as a fixed effect in the models.

Second, the narrowness of the confidence intervals probably stems from an underestimate of the uncertainty in parameter values. This is due to the fact that the observational errors are spatially and temporally correlated, and so we have fewer (effective) data points than we naively believe. This idea was first raised in [48], which discusses the fact that auto-correlated measurements, combined with an assumption of i.i.d. observational errors, can lead to an underestimation in parameter uncertainty in an ODE context. This auto-correlation is evident from the localized bursts of proliferation activity present in the experiments. A potential solution to this problem is to use a more realistic noise model where the observational noise is allowed to have spatial and temporal auto-correlation, by prescribing a correlation kernel with additional parameters to be fitted. This approach is discussed in [51] in the context of generalized linear models. Another approach that could be taken is to avoid making assumptions on the noise by replacing the likelihood function (which must be formulated with a noise model in mind) by a generalized profile-likelihood function, which relies less on the noise model, but instead must be calibrated by bootstrapping. The details of this approach are discussed in [52]. Alternatively, stochastic models might be more suitable in cases where process noise is significant. However, inference for stochastic models introduces computational challenges, making them unwieldy for many situations.

For the more complicated models, we observed a dependency of the parameter estimates on the experimental initial conditions. This is at odds with our assumption that the only mechanisms driving tissue expansion are cell motility and proliferation, and that the only factor modulating these mechanisms is cell density. A likely explanation for this dependency is that mechanical properties of the tissue may play a significant role in cell invasion. As such, an interesting avenue for future research entails including mechanical effects at the leading edge in the models.

Our findings reveal the pivotal impact of data resolution on parameter identifiability. As the amount of available data decreases, the confidence intervals for all parameters widen. However, certain parameters remain practically identifiable even at low data resolution, while others become non-identifiable if the data resolution is sufficiently low. This observation indicates that each model has its own minimum data requirement for practical identifiability, which tends to be higher for more complicated models.

We have also shown that the profile-likelihood method provides much more information about identifiability compared with methods based on the Fisher information matrix (FIM). A more traditional, and less computationally intensive, method for quantifying uncertainty in parameter estimates is to approximate the parameter distribution as a joint Gaussian distribution centred at the MLE, with covariance matrix derived from the FIM. However, this method essentially assumes a quadratic approximation to the likelihood function, which, as we have seen, can be far from valid in non-identifiable cases. The performance of such methods for uncertainty quantification is compared with profile likelihoods in [28].

Finally, we return to the problem of model selection. For most datasets, AIC and BIC select the same model as each other, which is usually the Richards or the Generalized Fisher models (see electronic supplementary material, section S3 for details). However, as we have seen, these models require more data to be identifiable, and they are also more sensitive to subtle changes in the data introduced by data processing (e.g. full density profile versus radial averaging). Simply increasing the penalty for model complexity in the AIC or BIC does not solve the problem, since identifiability cannot be properly accounted for by counting free parameters. Further, as we have seen, the Porous Fisher and Richards models have the same number of parameters, but Porous Fisher is more identifiable. Therefore, we argue that model selection methods should also take the amount of available data into account. For the problem considered in this work, more complicated models can be adopted if there are sufficient data to render them practically identifiable, whereas the Standard Fisher model should be favoured if the data resolution is low, or if we have only radially averaged data.

We would also like to address the distinction between parameter identifiability, which can be thought of as uncertainty quantification in parameter values, and prediction uncertainty, and the implications of each on model selection. Parameter uncertainty is not always mirrored in prediction uncertainty, which is, in fact, what drives practical identifiability issues. On the one hand, practical identifiability means the predictions of the model are sensitive to changes in parameter values, which, in turn, entails that the resulting posterior distributions and posterior predictive distributions are narrow. On the other hand, a practically non-identifiable model is insensitive to changes in parameter values, which implies that the predictions are robust to changes in parameter values. Therefore, it is difficult to determine, *a priori*, the relationship between identifiability and prediction uncertainty.

As such, both identifiability and prediction uncertainty are important in model selection, but the relative weight placed on each in making decisions depends on the particular focus of the modelling study. For example, if the goal of a modelling study is to provide quantitative insights into a particular biological mechanism, then that mechanism must be included in any candidate models considered. Identifiability, especially that of the parameters associated with the mechanism in focus, is hugely important in this case. If all models incorporating the necessary mechanisms are non-identifiable, then a practitioner should aim to use the models to design experiments that, when implemented, resolve the identifiability issues and thereby provide mechanistic insights. Furthermore, in cases where model parameters have concrete physical or biological meaning, it might be relevant to use these parameters in other studies. Precise parameter estimates are crucial for the validity of such approaches. However, for the purpose of investigating possible qualitative mechanisms to explain a biological phenomenon, a certain degree of non-identifiability is acceptable, and arguably desirable in certain biological applications, since it reflects robustness. Finally, while confidence in model predictions is clearly central for making predictions, identifiability remains important, since non-identifiability can lead the modeller to misplace their confidence in the reliability of model predictions in alternative scenarios.

In the future, we will extend our investigation to optimize experimental design with the aim to generate the most useful data for identifying model parameters. It would also be worthwhile to extend the scope of this investigation to stochastic models, which can be more suitable for systems with significant process noise. Finally, for cell invasion, it would be interesting to see if the addition of cell cycle data, such as those obtained via the fluorescence ubiquitin cell cycle indicator (FUCCI) reporter, can enhance parameter identifiability, or if the additional complexity introduced by including the cell cycle in the model hinders parameter identifiability instead.

## Data Availability

The data and code are available from the Zenodo digital repository: https://zenodo.org/records/10594283 [53]. The codes for performing the analysis are provided from the GitHub digital repository: https://github.com/liuyue002/woundhealing [54]. The animations of the datasets are provided from the GitHub repository: https://github.com/liuyue002/woundhealing/tree/public/experimental_data/animations [55]. Supplementary material is available online [56].

## Endnotes

1 We may also replace the subscript with the name of the replaced parameter for readability.

2 The profile-likelihood computation for the other four experiments could not be completed in reasonable time, due to difficulties in performing the optimizations during the computations for the profile likelihoods. We did not pursue this technical challenge further since it is beyond the scope of the paper.

3 A two-sample Kolmogorov–Smirnov test could not reject the null hypothesis that the MLEs for the experiments with different initial conditions come from the same distribution, with a *p*-value of 0.1074 for *D*_0_ and 0.5344 for *r*.

4 With a Kolmogorov–Smirnov test *p*-value of 0.0110.

5 The Kolmogorov–Smirnov test shows that the differences in *D*_0_ and *η* are significant, while the differences in *r* and *K* are not.
